# SARS-CoV-2 Omicron Variant in Patients With Chronic Myeloid Leukemia: A Retrospective Study

**DOI:** 10.7759/cureus.23863

**Published:** 2022-04-05

**Authors:** Elrazi A Ali, Ibrahim Khamees, Mohammad Abu-Tineh, Hana Qasim, Awni Alshurafa, Khalid Ahmed, Lujain Malkawi, Mohamed A Yassin

**Affiliations:** 1 Department of Internal Medicine, Hamad Medical Corporation, Doha, QAT; 2 Department of Medical Oncology, Hematology and BMT Section, National Center for Cancer Care and Research, Hamad Medical Corporation, Doha, QAT; 3 Department of Hematology and Medical Oncology, Hamad Medical Corporation, Doha, QAT; 4 Department of Internal Medicine, Jordan University of Science and Technology, Irbid, JOR; 5 Department of Hematology and Oncology, Hamad General Hospital, Doha, QAT

**Keywords:** chronic myelogenous leukaemia (cml), chronic myeloid leukemia (cml), covid-19, omicron variant, sars-cov-2

## Abstract

Background and aims

Coronavirus disease 2019 (COVID-19) is caused by a virus known as severe acute respiratory syndrome coronavirus 2 (SARS-CoV-2). Since the first pandemic wave, SARS-CoV-2 had developed significant changes and mutations that resulted in the emergence of different strains. Each strain varies in its virulence and disease severity. Most reports have shown that the Omicron variant causes mild illness. Little is known about the impact of Omicron in patients with chronic myeloid leukemia. We present patients with chronic myeloid leukemia who had infection with the Omicron variant of the SARS-CoV-2 and their outcomes.

Materials and methods

Retrospective data from the records of the National Center for Cancer Care and Research from December 20, 2021, to January 30, 2022. Participants were adults over the age of 18 years with Omicron infection who had been diagnosed with chronic myeloid leukemia according to World Health Organization classifications from 2008 and 2016.

Results

Eleven patients with chronic myeloid leukemia had Omicron infection. All patients had a mild disease according to the World Health Organization classification of COVID-19 severity. The majority of patients were young males.

Conclusions

In patients with chronic myeloid leukemia, infection with the Omicron variant of the SARS-CoV-2 usually results in mild disease not requiring hospitalization.

## Introduction

Since its first outbreak in Wuhan, China, near the end of 2019, severe acute respiratory syndrome coronavirus 2 (SARS-CoV-2) had quickly spread worldwide, resulting in global epidemics. The disease is more likely to be severe in patients with multiple comorbidities [1,2]. The presentation of SARS-CoV-2 infection is mainly with respiratory symptoms, but the involvement of other major organs like the liver, kidney, pancreas, and skin is also seen [3-5]. Others may present with complications like thrombosis [6]. SARS-CoV-2 continued to develop mutations in the spike proteins, which resulted in the emergence of different strains, which include Alfa, Beta, Gamma, Delta, and, finally, Omicron variants. Leukemia is a hematological disorder characterized by the unregulated production of white blood cells. The effect of SARS-CoV-2 on patients with chronic leukemia is variable. In patients with chronic lymphocytic leukemia, mortality is related to age, and the COVID infection is usually associated with increased white cell count [7,8]. Previous studies showed that chronic myeloid leukemia (CML) with SARS-CoV-2 infection had increased mortality. However, little is known about the effect of the Omicron strain of the SARS-CoV-2 virus on patients with CML. We are reporting patients with CML who developed infection with the Omicron variant of the SARS-CoV-2 virus. The data show the effect of the Omicron variant on this group of patients.

## Materials and methods

Study design

Retrospective data from records from the National Center for Cancer Care & Research (NCCCR) in Doha-Qatar, in the time period between December 20, 2021, and January 30, 2022. All patients are residing in the state of Qatar, with regular follow-up in NCCCR.

Inclusion and exclusion criteria

The study included 11 patients with CML who had Omicron infection. Patients were included if they met the two criteria. First, patients infected with the Omicron variant of SARS-CoV-2 were confirmed with a polymerase chain reaction. Second is adults over the age of 18 who had been diagnosed with CML according to WHO classifications from 2008 and 2016. Patients not meeting the inclusion criteria were excluded.

Ethical approval

The study was approved by the Medical Research Center with approval number MRC-04-22-150.

## Results

The majority of patients are young, with a mean age of 37.72 years (Table 1). Six patients had no comorbid condition, while five had medical conditions like diabetes, hypertension, and hypothyroidism. Ten out of 11 patients were males. All patients were in the chronic phase and on treatment with tyrosine kinase inhibitor (TKI) except one patient who was on hydroxyurea. All patients were previously diagnosed with CML for at least two years. All patients had no previous SARS-CoV-2 infection and all had a mild disease according to WHO criteria of COVID 19 severity [9]. No major complications were reported except in one patient who was unvaccinated and developed tumor lysis and acute kidney injury. The vaccines used were Pfizer-BioNTech (BNT162b2) and Moderna (mRNA-1273). Eight patients were vaccinated, seven patients received Pfizer, and one patient received Moderna. Three patients received three doses, and five patients had two doses. Only three patients required hospitalization and all are not directly related to SARS-CoV-2 infection. The first patient was admitted for blood transfusion because of anemia due to progression of CML and tested positive on routine testing on admission, and the second patient was hospitalized due to nausea, vomiting, and acute kidney injury requiring intravenous fluids. The last patient had contact with a positive patient and was shifted from home isolation to the COVID facility.

**Table 1:** Characteristics of patients with CML who had Omicron infection CKD: chronic kidney disease; PMH: past medical history; CML: chronic myeloid leukemia; MPN, myeloproliferative neoplasm; AKI, acute kidney injury; TLS, tumor lysis syndrome.

Age	Gender	PMH or comorbidities	Duration of disease (MPN)	Active treatment for CML	Vaccination status	Vaccine type received	Previous COVID-19 infection	Previous thrombosis or hemorrhage with COVID -19	Other complications with previous COVID-19 infection	Omicron COVID-19 severity	Hospitalization	Complications and outcome
32	Male	None	1 year	Ponatinib	2 doses	Pfizer	None	None	None	Mild	Yes	Good outcome
45	Male	Hypothyroidism	5 years	Imatinib, hydroxyurea	None	None	None	None	None	Mild	Yes	AKI, TLS
28	Male	None	2 years	Dasatinib	2 doses	Moderna	None	None	None	Mild	None	Good outcome
38	Female	None	4 years	Hydroxyurea	None	None	None	None	None	Mild	Yes	Good outcome
42	Male	None	5 years	Imatinib	2 doses	Moderna	None	None	None	Mild	None	Good outcome
42	Male	None	7 years	Imatinib	3 doses	Pfizer	None	None	None	Mild	None	Good outcome
45	Male	Hypertension, asthma	2 years	Imatinib	2 doses	Pfizer	None	None	None	Mild	None	Good outcome
52	Male	Hypertension, diabetes, dyslipidemia	6 years	Dasatinib	None	None	None	None	None	Mild	None	Good outcome
65	Male	Osteomyelitis, diabetes, dyslipidemia, CKD, peripheral vascular disease	10 years	Nilotinib	None	None	None	None	None	Mild	None	Good outcome
45	Male	None	3 years	Nilotinib	3 doses	Pfizer	None	None	None	Mild	None	Good outcome
61	Male	Hypercholestrelemia	5 years	Imatinib	3 doses	Pfizer	None	None	None	Mild	None	Good outcome

## Discussion

CML is a hematologic disorder with the overproduction of mature granulocytes. It can present with symptoms or may be discovered during investigations for other complaints [10]. Others may present with ophthalmologic or urologic complications [11]. The cornerstone for the treatment of CML in the chronic phase is TKI of a different generation [12,13]. Like other patients with malignancies, patients with CML are susceptible to infection; this includes infections like TB hepatitis [14,15], as well as infections like SARS-CoV-2. SARS-CoV-2 is a respiratory virus that has caused the most infections worldwide. Omicron is distinguished from other SARS-CoV-2 strains by its high replication rate [16], ability to evade the humoral immune response, and high reinfection rate [17]. This resulted in Omicron to cause a massive pandemic that affected millions of individuals.

Data regarding the effect of SARS-CoV-2 infections in patients with CML are scarce. In patients in the chronic phase of CML who are on TKI, infection with SARS-CoV-2 was not associated with worse outcomes or increased mortality [18,19]. It was thought that TKI might have a protective effect on these patients against developing worse outcomes [19]. However, a large-scale study showed a mortality rate of 13.7% in CML patients with COVID-19 infection [20]. All these data were in patients with previous strains of the SARS-CoV-2 virus. The data regarding COVID-19 infection in patients with CML are scarce, and they are mainly on the wild (alpha) variant. With the Omicron variant, our reported cases showed that CML patients presented with mild illness. 

Infection with Omicron strain complicates our understanding of SARS-CoV-2; it makes the effect of the infection on leukemia patients more complex, as currently, most of the population, including patients with CML, have received vaccinations. Additionally, patients with leukemia are immunocompromised, and the vaccine's effectiveness in this group of patients is expected to be lower than in other populations [21]. For this reason, it is recommended to have a third dose to boost the immunity against Omicron, which has partial immunity with two doses of vaccines. Moreover, the SARS-CoV-2 infection was mild, and no serious complications were reported except in unvaccinated patients. This may be due to the fact that Omicron causes milder disease in this group of patients than in the general population, or it is due to the protective effect of vaccination that resulted in milder disease and low hospitalization. 

The main limitation of the study is the small sample size. The data showed 11 patients, and all had mild disease and good outcomes. To generalize the result, a powerful study is needed with large sample size. With the limited number of patients, it is difficult to understand the factors related to disease severity in patients with CML and to evaluate the effect of the different medications on the patient outcome.

## Conclusions

The reported data suggest that the Omicron variant of SARS-CoV-2 usually presents with mild illness in patients with CML. This looks similar to the general population who are not immunocompromised, where Omicron is generally milder and requires no hospital admission. However, a large-scale study is required to better understand the Omicron effect in patients with CML.
